# Combined Inhibition of Polyphenol Oxidase by Oxyresveratrol and Epigallocatechin Gallate: A Natural Anti-Browning Strategy for Fresh-Cut Pears

**DOI:** 10.3390/foods15172960

**Published:** 2026-08-23

**Authors:** Ruobing Liu, Zhiqiang Ren, Nuoran Rong, Jingyu Wei, Xiaoyan Zhang, Yong Peng

**Affiliations:** College of Food Science and Engineering, Shandong Agricultural University, 61 Daizong Road, Tai’an 271018, China; lrb5720724@163.com (R.L.); m13953017270@163.com (Z.R.); rongrn678@163.com (N.R.); wjydpl@163.com (J.W.); zhangxiaoyan@sdau.edu.cn (X.Z.)

**Keywords:** inhibition rate, molecular docking, conformational change, enzymatic browning, fresh-cut produce

## Abstract

Polyphenol oxidase (PPO) is a key enzyme responsible for enzymatic browning in fresh-cut fruits and vegetables, severely compromising their quality and shelf life. This study aimed to investigate the combined inhibitory mechanism of oxyresveratrol (OXY) and epigallocatechin gallate (EGCG) on PPO through multi-spectroscopic analyses, molecular docking, TEM, and XRD, with the goal of developing a natural and effective anti-browning strategy for fresh-cut fruits. The results showed that the optimal combined effect was achieved at an OXY:EGCG ratio of 1:2, where the inhibition rate was significantly enhanced by 43.72% and 14.36% compared to using OXY or EGCG single treatment, respectively. The combined treatment exhibited enhanced chelation capacity of copper ion and DPPH radical scavenging activity, and enhanced hydrogen-bonding interactions while lowering binding energy, exhibiting characteristics of mixed inhibition kinetics. Structural characterization showed that the combined treatment drastically reduced the enzyme’s fluorescence intensity to 39.81% of that of the native enzyme, induced rearrangements in α-helix and random coil structures, triggered obvious protein aggregation, and weakened the intensity of crystal diffraction peaks. Importantly, the combined treatment effectively delayed browning in fresh-cut pear slices, demonstrating its practical application potential. These findings provide a promising natural combined approach for controlling enzymatic browning and extending the shelf life of fresh-cut produce.

## 1. Introduction

Fresh-cut fruits and vegetables are highly favored by consumers mainly due to their consumption convenience, while visual freshness acts as one of the critical factors for consumers’ purchasing decisions. Nevertheless, mechanical damage during processing readily triggers enzymatic browning, which causes color and flavor deterioration, and consequently shortens shelf life and reduces commercial value [1]. Enzymatic browning of fresh-cut produce involves reactive oxygen metabolism and the regulatory roles of browning-related enzymes such as polyphenol oxidase (PPO), peroxidase (POD) and phenylalanine ammonia-lyase (PAL). Additionally, it is closely related to the intrinsic antioxidant ability of the produce itself. Research indicates that the most key factor in browning is the catalytic action of polyphenol oxidase (PPO). This enzyme, a copper-containing oxidoreductase widely present in fruits and vegetables, features a molecular structure comprising an N-terminal leader peptide, a central copper ion active site, and a C-terminal hydrophobic region [2,3]. When fruits and vegetables undergo mechanical damage, cold stress, or pathogen infection, cellular integrity is disrupted. This allows PPO to come into full contact with phenolic substrates, initiating a catalytic reaction in which monophenols are first hydroxylated into ortho-diphenols, then oxidized into ortho-quinones and further polymerized into brownish-black substances [4,5].

The methods of controlling the browning and PPO activity include physical, chemical, biological and combined means. Among them, plant extract indicated good preservation effect and high safety, particularly some polyphenolic substances, which can inhibit PPO activity through non-covalent interactions and strong antioxidant to alter protein structure and function [6]. The study found that oxyresveratrol (OXY) is a natural polyphenolic compound widely found in plants such as grapes, polygonum multiflorum and mulberries, exhibiting excellent antioxidant ability, anti-inflammatory and enzyme-inhibiting activities [7]. Research has confirmed that OXY significantly inhibited PPO activity, primarily through chelation with copper ions in the enzyme’s active site, thereby disrupting the catalytic site [8]. Additionally, Zeng et al. [9] revealed that OXY altered the microenvironment surrounding the enzyme’s catalytic domain and fluorescent group, subsequently affecting enzyme structure. Furthermore, OXY can bind to amino acid residues via hydrogen bonds, altering the active site and thereby inhibiting enzyme activity [10]. In addition, epigallocatechin gallate (EGCG), the most abundant catechin in green tea, is also a natural polyphenol with potent antioxidant ability [11]. In recent years, its inhibitory effect on PPO has become a research focus. Studies indicate that the hydroxyl group of catechins possesses electron-donating reducing capabilities, and could block melanin polymerization by reducing the orthoquinone products catalyzed by PPO [12]. Concurrently, EGCG could occupy the active site position, altering the enzyme’s spatial conformation through non-covalent interactions such as hydrogen bonding and hydrophobic interactions with amino acid residues, thereby reducing enzyme activity [13].

Although the individual mechanisms by which OXY or EGCG inhibit PPO activity have been partially elucidated, their application as inhibitors are often constrained by low inhibitory efficacy and poor stability under physiological conditions. Consequently, combining the two compounds to achieve combined effects holds considerable practical value. However, systematic investigations into their combined impact on PPO activity and structure remain lacking. Other studies show that the combined effects of different polyphenols (sanggenone C, oxyresveratrol, catechin and L-epicatechin) exert a greater influence on the hydrophobic microenvironment surrounding the tyrosinase catalytic center, and the combined effects are closely related to polyphenol concentration [10]. Furthermore, under high hydrostatic pressure, EGCG and ferulic acid form stable structures with the PPO active site through hydrogen-bonding interaction, thereby inhibiting PPO activity [14].

Therefore, this study systematically elucidates the combined mechanism of OXY and EGCG against PPO, spanning from macro-scale activity to microstructural changes. Initially, combined effects are validated through enzyme activity, antioxidant assays and kinetic analysis coupled with molecular docking. Subsequently, spectroscopic techniques, TEM, and XRD characterize the disruption of the enzyme spatial structure. This work aims to provide a theoretical foundation for developing efficient browning control technologies and offers novel insights for the industrial application of green preservation strategies in fresh-cut fruits and vegetables.

## 2. Materials and Methods

### 2.1. Materials

PPO (from mushrooms), OXY and EGCG used in this study were purchased from Shanghai Yuanye Biotechnology Co., Ltd., Shanghai, China. Reagents including catechol, levodopa, Phosphate-buffered saline (disodium hydrogen phosphate and sodium dihydrogen phosphate), pyridine, copper sulfate, DPPH, anhydrous ethanol and uranyl acetate were purchased from Tianjin Kaitong Chemical Reagent Co., Ltd., Tianjin, China. Reagents such as resorcinol purple were purchased from Shanghai McLean Biochemical Technology Co., Ltd., Shanghai, China. Polyvinylpolypyrrolidone (PVPP) was purchased from Beijing Solarbio Science & Technology Co., Ltd., Beijing, China.

### 2.2. PPO Inhibition Rate Assay

All combined treatments in this study were prepared by mixing OXY stock solution and EGCG stock solution at a fixed volume ratio of 1:2. The experimental method was adapted from the protocol described by Zhang et al. [8]. Pure PPO enzyme was dissolved in 0.10 mol/L phosphate buffer (pH 6.8) to prepare an enzyme solution with an activity of no less than 150 U/mL. A 3 mL reaction system was composed of 0.75 mL of 0.02 mol/L substrate (either catechol or levodopa), 0.75 mL of enzyme solution (0.02 mg/mL), and 1.5 mL of inhibitor. Specifically, the treatments included single OXY, single EGCG treatment, and combined inhibitor mixtures with OXY:EGCG ratios of 1:1, 1:2, 1:3, 1:4, 2:1, 3:1, and 4:1. The absorbance (*A_i_*) was measured at 420 nm when catechol was used as the substrate and at 470 nm when levodopa was used as the substrate. For the control group, a phosphate buffer solution was used instead of the inhibitor. Each treatment was conducted in three replicates and the average value was calculated. When catechol was employed as the substrate, the concentrations of OXY and EGCG were 0.21 mmol/L and 0.13 mmol/L, respectively. Based on preliminary experimental findings, the affinity between PPO and different substrates required different inhibitor concentrations. Therefore, when using levodopa as the substrate, the concentrations were set at 2.00 μmol/L for OXY and 1.00 mmol/L for EGCG.

PPO inhibition rate (%) = (1 − *A_i_*/*A*_0_) × 100% (1)

In the formula: *A_i_*: the absorbance of the treatment group; *A*_0_: the absorbance of the control group.

### 2.3. Evaluation of the Combined Coefficient of OXY and EGCG

To compare the combined effects of OXY and EGCG at different ratios under a fixed concentration, this study adopted a simplified combined coefficient λ as a preliminary screening index, following the concept of classical Tallarida’s evaluation methods [15]. This coefficient was used solely for a direct comparison of the combined inhibitory potency across different ratio groups at the same concentration level. According to this simplified criterion, λ > 1 indicates that the combined effect is greater than the mean of the individual treatments, and λ > 1.2 suggests a relatively pronounced enhancement.

*λ* = 2*C*/(*A* + *B*) (2)

In the formula: *A*: the PPO inhibition rate with single OXY; *B*: the PPO inhibition rate with single EGCG; *C*: the PPO inhibition rate under combined treatment.

### 2.4. Copper Ion Chelation Ability

Based on the method described by Zhang et al. [8], 1 mL of CuSO_4_ (2 mmol/L) solution and 1 mL of pyridine were mixed and added to 0.1 mL of 0.324 mmol/L pyrocatechol violet and 2 mL of inhibitor solution (OXY, EGCG, and combined inhibitors at different ratios as in Section 2.2). After mixing thoroughly, the mixture was allowed to stand at room temperature for 10 min, and the absorbance at 632 nm was measured. The phosphate buffer solution instead of the inhibitor was used as control group. Each treatment was repeated three times, and the average value was recorded.

Copper ion chelation ability (%) = (1 − *A_i_*/*A*_0_) × 100% (3)

In the formula: *A_i_*: the absorbance of the treatment group; *A*_0_: the absorbance of the control group.

### 2.5. DPPH Radical Scavenging Ability

Based on the method of Shi et al. [11], in a centrifuge tube, 2 mL of inhibitor (OXY, EGCG and combined inhibitors at different ratios) was added, followed by 2 mL of 0.2 mmol/L DPPH–ethanol solution. After mixing thoroughly, the mixture was reacted at room temperature in the dark for 30 min. The absorbance at 517 nm was determined and anhydrous ethanol without inhibitor was used as the control group. Each treatment was replicated three times, and the average value was recorded.

DPPH radical scavenging rate (%) = (1 − *A_i_*/*A*_0_) × 100% (4)

In the formula: *A_i_*: the absorbance of the treatment group; *A*_0_: the absorbance of the control group.

### 2.6. Molecular Docking

The crystal structure of polyphenol oxidase (PPO, PDB ID: 2Y9W) was downloaded from the RCSB Protein Data Bank (https://www.rcsb.org/). The 3D structures of oxyresveratrol (OXY) and epigallocatechin gallate (EGCG) were obtained from the PubChem database (https://pubchem.ncbi.nlm.nih.gov/). The receptor (PPO) was processed by dehydration and hydrogenation, and the ligands (OXY and EGCG) were hydrogenated prior to export. A total of 20 independent molecular docking simulations were performed using AutoDock Vina (version 1.25), and the corresponding PDBQT files were generated. These files were then converted to PDB format using OpenBabel software (version 3.1.1). Visualization and analysis were carried out using PyMOL (version 3.1.5.1), and the optimal docking conformation was determined according to the binding energy values, thereby establishing a 3D molecular docking model.

### 2.7. Inhibition Kinetics

The substrate (0.75 mL) contained levodopa at four different concentrations (0.03 mol/L, 0.02 mol/L, 0.015 mol/L and 0.01 mol/L) was selected based on the method of Zhang et al. [8]. A 3 mL reaction system was prepared by mixing 0.75 mL of enzyme solution (0.02 mg/mL) and 1.5 mL of OXY + EGCG combined inhibitor (1:2, *v*/*v*) with three different concentrations (10.00 μmol/L OXY + 2.00 mmol/L EGCG, 5.00 μmol/L OXY + 1.00 mmol/L EGCG, and 2.50 μmol/L OXY + 0.50 mmol/L EGCG). The mixture was incubated at 30 °C for 30 min, and its absorbance was measured at 470 nm. For the control group, a phosphate buffer solution was used instead of the inhibitor. Each treatment was conducted in three replicates and the average value was recorded. A double reciprocal plot was constructed to identify the inhibition type of the OXY and EGCG combined treatment.

### 2.8. Full-Wavelength UV Spectroscopy

Based on the method described by Xu et al. [16], a full-wavelength UV scanner (UV-2600i, Shimadzu Instruments Co., Ltd., Kyoto, Japan) was employed to determine the UV-visible absorption spectra of PPO under different treatment conditions. The reaction system was composed of enzyme solution (0.02 mg/mL), phosphate buffer (0.1 mol/L, pH 6.8), and inhibitor solution. Absorption spectra were measured at a wavelength range of 250–600 nm for various samples, including pure PPO solutions, individual and combined solutions of OXY and EGCG, and PPO solutions treated with individual or combined solutions. The concentrations of OXY and EGCG were set at 50.00 μmol/L and 15.00 μmol/L, respectively.

### 2.9. Fluorescence Spectra

The experimental procedure was adapted from Tian et al. [13] with minor adjustments. A fluorescence spectrophotometer (LF-1701009, Thermo Fisher Scientific, Waltham, MA, USA) was used to measure the endogenous fluorescence emission spectra of PPO treated with the OXY and EGCG combined solution. The reaction system consisted of enzyme solution (0.2 mg/mL), inhibitor solution and phosphate buffer (0.1 mol/L, pH 6.8), with the control group using phosphate buffer instead of the inhibitor. The excitation wavelength was set to 280 nm with a scanning range of 300–400 nm, and both the excitation and emission slit widths were set at 5 nm. The concentrations of OXY and EGCG were 10.20 μmol/L and 25.00 μmol/L, respectively, in the measurement.

### 2.10. Circular Dichroism Spectroscopy (CD)

The experimental method was adapted from Cheng et al. [17] with minor modifications. The reaction system consisted of enzyme solution (0.2 mg/mL), inhibitor solution and phosphate buffer (0.1 mol/L, pH 6.8). The concentrations of OXY and EGCG inhibitor were set at 10.00 μmol/L and 30.00 μmol/L, respectively, and phosphate buffer was used as the control group. The scanning wavelength range was set to 190–250 nm, with a bandwidth of 1 nm and a scanning speed of 50 nm/min. Background correction was conducted using 0.1 mol/L phosphate buffer (pH 6.8). Spectral data were expressed as molar circular dichroism (mdeg) and the relative proportions of each component in the PPO secondary structure were calculated.

### 2.11. Transmission Electron Microscopy (TEM)

The experimental procedure was carried out referring to the method described by Du et al. [18] with minor modifications. A 5 μL of sample obtained from the pre-prepared reaction mixture contained enzyme solution (1 mg/mL) and inhibitor solutions (OXY, EGCG, OXY + EGCG at a 1:2 ratio) was dried on a copper mesh. After blotting the excess liquid with filter paper, the sample was stained with 2.0% uranyl acetate for 60 s, followed by 30 s of staining solution treatment. Subsequently, the samples were observed under a transmission electron microscope (Talos L120C, Thermo Fisher Scientific, Waltham, MA, USA) at an operating voltage of 120 kV.

### 2.12. X-Ray Diffraction (XRD)

The method described by Jin et al. [19] was followed with minor modifications. Samples containing enzyme solution (1 mg/mL) and inhibitor solutions (OXY, EGCG, and OXY + EGCG (1:2)) were lyophilized and placed in an X-ray diffractometer (Smartlab SE, Rigaku Corporation, Tokyo, Japan). Scanning was performed over the 2θ range of 5–80° at a scanning speed of 24 °/min.

### 2.13. Application on Fresh-Cut Pear

Snow pears were purchased from Aolaifeng Market, Tai’an City, China. All fruits were harvested at a commercial maturity, with uniform size without diseases or physical damage, and had not been stored before the experiment. Following thorough washing and peeling, the pears were cut into slices (3 mm thickness). Five treatment groups were set up, and one group was directly placed into sealable bags immediately after slicing, whereas the other four groups were separately immersed in 500 mL of distilled water (CK), 0.2 mmol/L OXY solution, 0.2 mmol/L EGCG solution and OXY + EGCG mixed solution (1:2 ratio, *v*/*v*) for 5 min. After soaking, the pear slices were blotted dry with absorbent paper and placed into polyethylene (PE, size:120 mm × 170 mm, thickness: 0.2 mm) sealable bags for storage. A colorimeter (CR-400, Jinan Kodar Industrial Co., Ltd., Jinan, China) was employed to determine the surface color changes at 0, 24, and 48 h, with three replicates for each treatment. The browning evaluation method was adopted from Zhang et al. [8]: browning area over 50% was categorized as extremely severe browning (5 points), 20–50% as severe browning (4 points), 5–20% as moderate browning (3 points), 0–5% as mild browning (2 points), and no browning (1 point).

PPO activity was assayed based on the methods of Zhang et al. [8] with slight modifications. Crude enzyme extracts were prepared by homogenizing 1.0 g of freeze-dried pear powder with 0.05 g of polyvinylpolypyrrolidone (PVPP) in 3 mL of 0.1 mol/L phosphate buffer (pH 6.8). The homogenate was centrifuged at 10,000 r/min for 15 min at 4 °C, and the supernatant was collected for analysis. Enzyme activity was measured spectrophotometrically using catechol as the substrate. The reaction system contained 1.5 mL of phosphate buffer, 0.75 mL of 0.1 mol/L catechol, and 0.75 mL of the enzyme extract. Phosphate buffer was used as the blank control instead of the enzyme solution. The increase in absorbance at 420 nm was recorded and one unit of PPO activity (U) was defined as the amount of enzyme causing a 0.01 change in absorbance per minute.

### 2.14. Statistical Analysis

At least three replicates were conducted for all experimental measurements of each treatment, and the resulting data were expressed as mean ± standard deviation. Statistical analysis was implemented with SPSS 26.0 software, one-way analysis of variance (ANOVA) was applied first, followed by Duncan’s post hoc test to identify significant differences among groups with the significance level set at *p* < 0.05. The experimental data were plotted and visualized by means of Excel 2021 and Origin 2022 software packages.

## 3. Results and Discussion

### 3.1. Effects of OXY and EGCG on PPO Inhibition Rate, Cu^2+^ Chelation Ability and DPPH Radical Scavenging Rate

**Figure 1:**
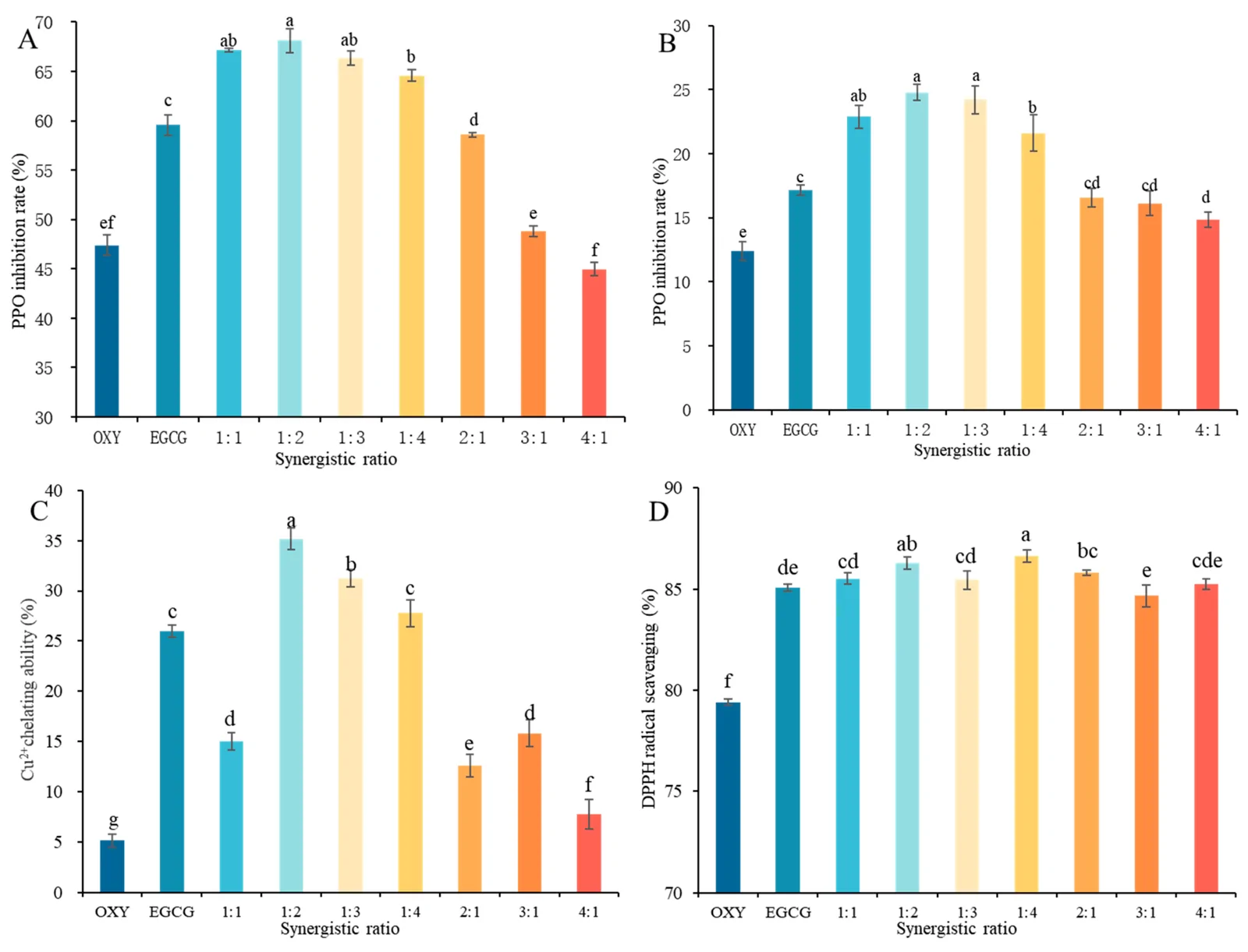
Effects of combined treatment with oxyresveratrol (OXY) and epigallocatechin gallate (EGCG) on PPO inhibition rate ((**A**) substrate is catechol; (**B**) substrate is levodopa), Cu^2+^ chelating ability (**C**) and DPPH radical scavenging rate (**D**). Different lowercase letters indicate significant differences among groups (*p* < 0.05).

As shown in Figure 1A, the PPO inhibition rates of single OXY and EGCG treatment were 47.39% and 59.56%, respectively, when using catechol as the substrate. The enhanced inhibitory effects were observed in combined treatment group. The optimal combined ratio of OXY and EGCG was 1:2, achieving a PPO inhibition rate of 68.11%, representing increases of 43.72% and 14.36% compared to OXY and EGCG single treatment, respectively. When the substrate was changed to levodopa (Figure 1B), the 1:2 combined ratio remained the optimal results, and the inhibition rates were 99.98% and 44.49% higher than those achieved by single OXY and EGCG treatment, respectively. In this study, inhibitor concentrations were set to achieve equivalent inhibition rates based on the pre-experiment results and substrate affinity. In the catechol system, the strong substrate binding resulted in millimolar levels for both OXY and EGCG. However, in the levodopa system, compared to EGCG, the small molecular size of OXY allowed to access the active site more readily, achieving comparable inhibition at micromolar concentrations.

Furthermore, the combined coefficients exceeded 1 for ratios ranging from 1:1 to 2:1 with catechol as the substrate, and the highest combined coefficient was 1.27 at the 1:2 ratio (Table 1). For levodopa as the substrate, all combined coefficients exceeded 1, reaching 1.68 at the 1:2 ratio. This indicated that the effect of OXY and EGCG on PPO activity was combined rather than additive.

The action mechanism of inhibitor on PPO activity can be preliminarily elucidated by copper ion chelation ability, serving as one method to characterize combined effects [20,21]. As shown in Figure 1C, combined treatments at ratios of 1:2, 1:3, and 1:4 effectively chelated copper ions. The 1:2 ratio of OXY and EGCG achieved a Cu^2+^ chelation rate of 35.18%, which was 584.44% and 35.31% higher than the rates achieved by single OXY and EGCG treatment. Furthermore, this study found that treatment groups with higher EGCG content in the ratios exhibited higher Cu^2+^ chelation abilities, suggesting EGCG plays a primary role in the combined effect. Some studies also indicated that oxyresveratrol [8], ursolic acid [22], chicory furaneol [23], and glutamic acid [21] inhibited PPO activity by enhancing copper ion chelation ability. This was similar with our results.

As shown in Figure 1D, the DPPH radical scavenging rate of the single EGCG treatment was significantly higher than that of the single OXY treatment. All combined treatment groups exhibited higher DPPH radical scavenging rates, and the rates of the 1:2 and 1:4 combined treatment groups reached 86.30% and 86.64%, respectively, indicating that combined treatment enhanced the antioxidant capacity of the reaction system. In other studies, Zhao et al. [24] found that the combined treatment of EGCG and pullulan polysaccharide significantly increased DPPH radical scavenging rate and antioxidant ability in passion fruit. This may be attributed to the fact that phenolic hydroxyl groups are generally considered to scavenge radicals by providing electrons to reduce reactive oxygen species. OXY and EGCG contained four and eight phenolic hydroxyl groups, respectively, thereby combined treatment increased the total phenolic hydroxyl content in the reaction system and enhanced antioxidant ability [25]. Enhanced antioxidant ability could reduce enzymatically generated quinones back to phenolic compounds, blocking their further polymerization and melanin formation, thereby mitigating browning [26].

### 3.2. Inhibition Kinetic

**Figure 2:**
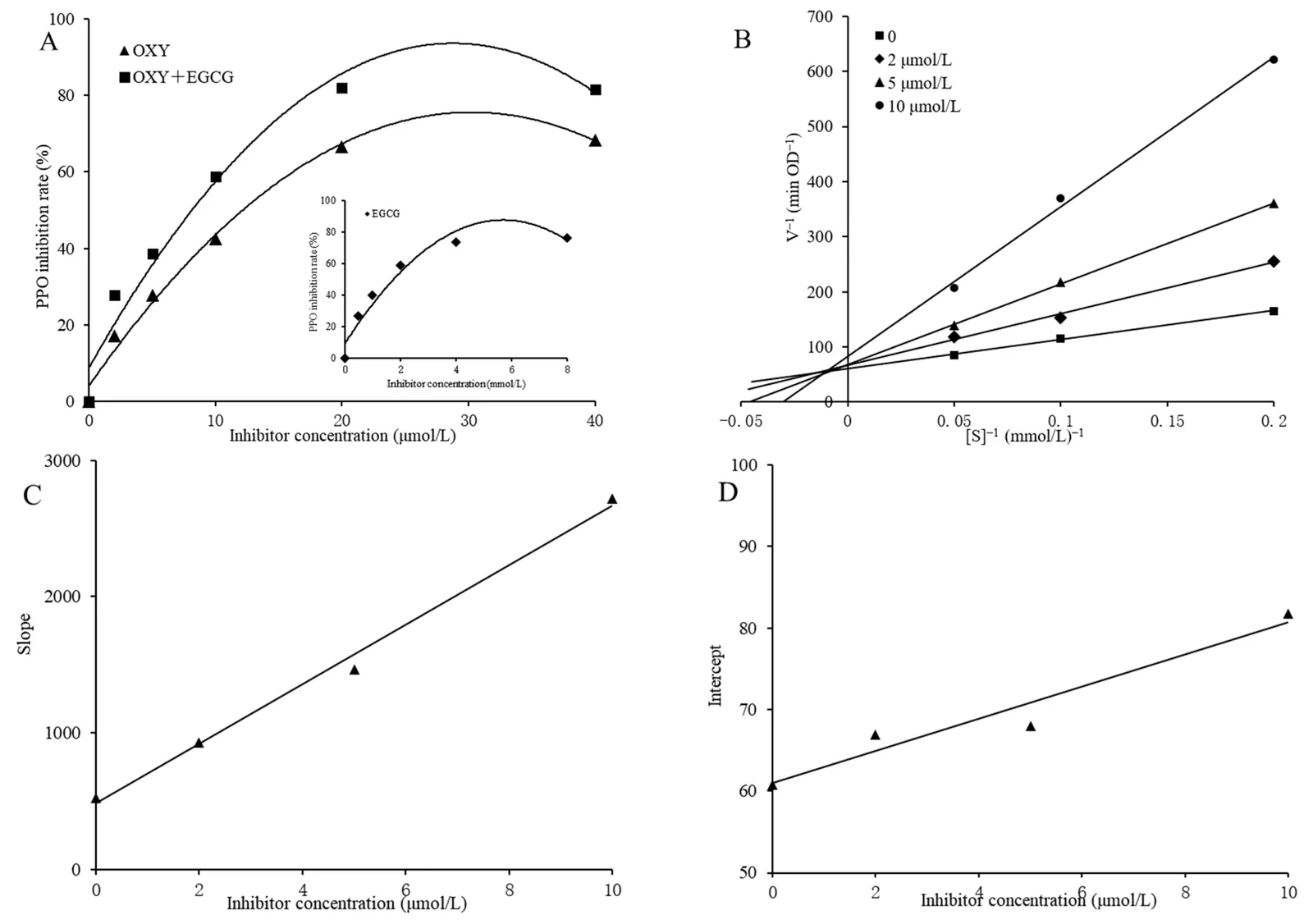
Effects of combined treatment with OXY and EGCG (1:2) on PPO inhibition rate (**A**), double reciprocal curve (**B**), slope plot (**C**), and intercept plot (**D**).

As shown in Figure 2A, the PPO inhibition rate increased rapidly at low inhibitor concentrations and gradually slowed with the increasing concentrations. The IC_50_ values for OXY and EGCG single treatment were 12.14 μmol/L and 1.75 mmol/L, respectively, while the combined treatment group (1:2 for OXY and EGCG, *v*/*v*) exhibited an IC_50_ of 8.14 μmol/L (the converted equivalent concentration of OXY), which was significantly lower than those of the single groups. The double reciprocal plot (Figure 2B) revealed that the intersection point for the combined inhibitor lay in the second quadrant based on the analysis of concentration and reaction rate. With the increasing combined inhibitor, the Vmax value continuously decreased while the Km value continuously increased, consistent with mixed-type inhibition. This showed that PPO might possess multiple binding sites for OXY and EGCG. This was similar with the inhibition types reported by Tian et al. [27] and Sae-leaw et al. [28] for EGCG on polyphenol oxidase in mushroom and Pacific white shrimp, respectively. Plotting the combined concentration against the slope and intercept of each linear regression (Figure 2C,D) yielded the inhibition constants KI for free PPO and KIS for the enzyme–substrate complex at 2.19 μmol/L (R^2^ = 0.9938) and 30.81 μmol/L (R^2^ = 0.9442), respectively. This indicated that the combined inhibitor could bind to both the free enzyme, affecting enzyme–substrate affinity and the enzyme–substrate complex, thus disturbing the catalytic degradation of the complex. Comparatively, its binding affinity for the free enzyme is relatively stronger. Song et al. [12] similarly observed KIS > KI when studying EGCG and GCG (gallocatechin gallate) inhibition of tyrosinase, indicating a greater tendency for the inhibitor to bind to tyrosinase. This binding preference was further corroborated across different enzyme sources and inhibitor systems, Zhang et al. [29], using L-cysteine as an inhibitor against pine-needle PPO, also observed KIS > KI, confirming its higher affinity for the free enzyme. Furthermore, Yu et al. [30] found that a polyphenol mixture composed of quercetin, cinnamic acid, and ferulic acid exhibited a significant combined inhibitory effect on tyrosinase, with an inhibition constant (KI) lower than that of any single component. This confirmed the presence of combined interactions among polyphenols.

### 3.3. Molecular Docking

**Figure 3:**
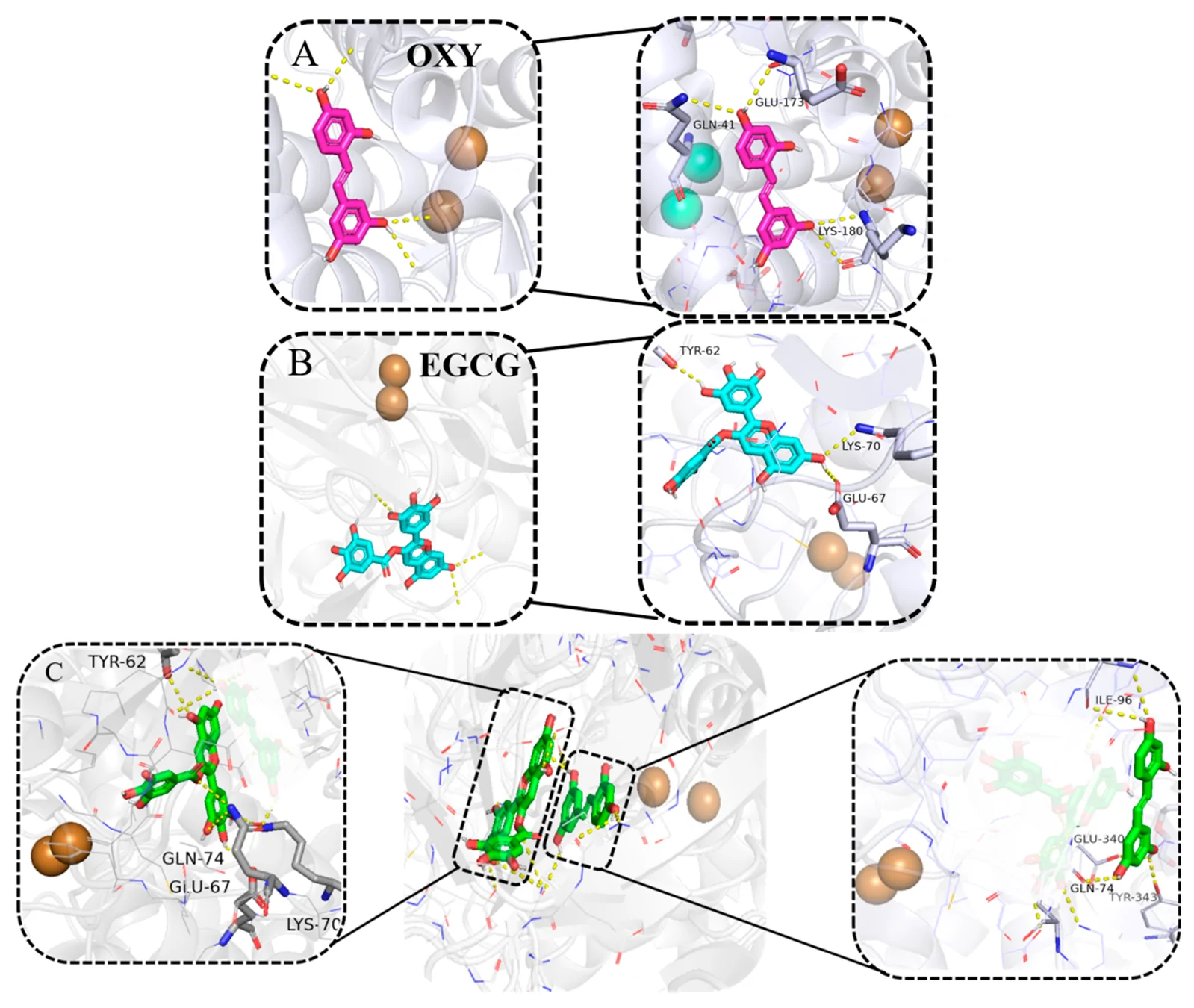
Three-dimensional molecular docking plots of PPO (2Y9W) by single OXY treatment (**A**), single EGCG treatment (**B**) and OXY and EGCG combined treatments (**C**).

Molecular docking predicts the binding sites and interaction types between small-molecule inhibitors and proteins [31]. As shown in Figure 3A,B, when OXY and EGCG were individually docked with the PPO (2Y9W) protein, their binding energies were −8.09 kcal/mol and −10.41 kcal/mol, respectively. OXY formed four hydrogen bonds with Lys180, Glu173 and Gln41 of PPO, while EGCG formed three hydrogen bonds with Glu67, Lys70, and Tyr62. When both were docked simultaneously with PPO (Figure 3C), the binding energy was −16.63 kcal/mol. The OXY-EGCG-PPO complex exhibited ten hydrogen bonds, with OXY forming five hydrogen bonds with PPO residues Ile96, Glu340, Tyr343, and Gln74, with bond lengths of 2.7, 3.1, 2.7, 2.2, and 2.3 Å, respectively. EGCG formed five hydrogen bonds with Gln74, Glu67, Lys70, and Tyr62 of PPO, with bond lengths of 3.4, 3.5, 2.3, 2.6, and 2.5 Å, respectively (Table 2). Compared to single treatments with OXY or EGCG, the binding energies decreased by 8.54 and 6.22 kcal/mol, respectively, indicating improved conformational stability. Regarding hydrogen bonding, the number of bonds formed in simultaneous docking exceeded that of single-agent docking. These results indicated that combined treatment led to lower binding energies and more hydrogen bonds than single treatment. This not only enhanced binding affinity between the inhibitor and enzyme, but also induced conformational changes in the enzyme through the formation of more stable complexes. As the primary binding force, hydrogen bonds played a crucial role in ligand recognition and complex conformation stability. Chen et al. [32] observed that the binding affinity between saponins of *Polygonatum sibiricum* and target enzyme (tyrosinase, elastase and hyaluronidase) molecules were below −7.00 kcal/mol, indicating stable and strong complex formation. In studies of blackberry anthocyanin complexes with tyrosinase, combined docking exhibited significantly lower binding energies than single-component docking, and a greater number of hydrogen bonds formed [33]. This substantially influenced binding efficiency and stability, consistent with our findings. Furthermore, Tian et al. [27] found that EGCG interacted with the PPO surface, and affecting its secondary structure. It was noteworthy that pentagalloylglucose (PGG), a polyphenolic tyrosinase inhibitor with strong Cu^2+^ chelation capacity, also forms hydrogen bonds predominantly with non-histidine residues (e.g., Glu-173, Lys-158) in molecular docking [34]. The discrepancy between the in vitro Cu^2+^ chelation assay (Section 3.1) and the docking results in this study may arise from the different chemical states probed by the two methods: the chelation assay uses free Cu^2+^ in solution, whereas in the docking simulation, the binuclear copper ion is fully coordinated by endogenous histidine residues and buried inside the intact enzyme active pocket; under the rigid receptor condition, the ligand cannot directly access the dicopper center.

### 3.4. UV Spectral Analysis of OXY and EGCG on PPO

**Figure 4:**
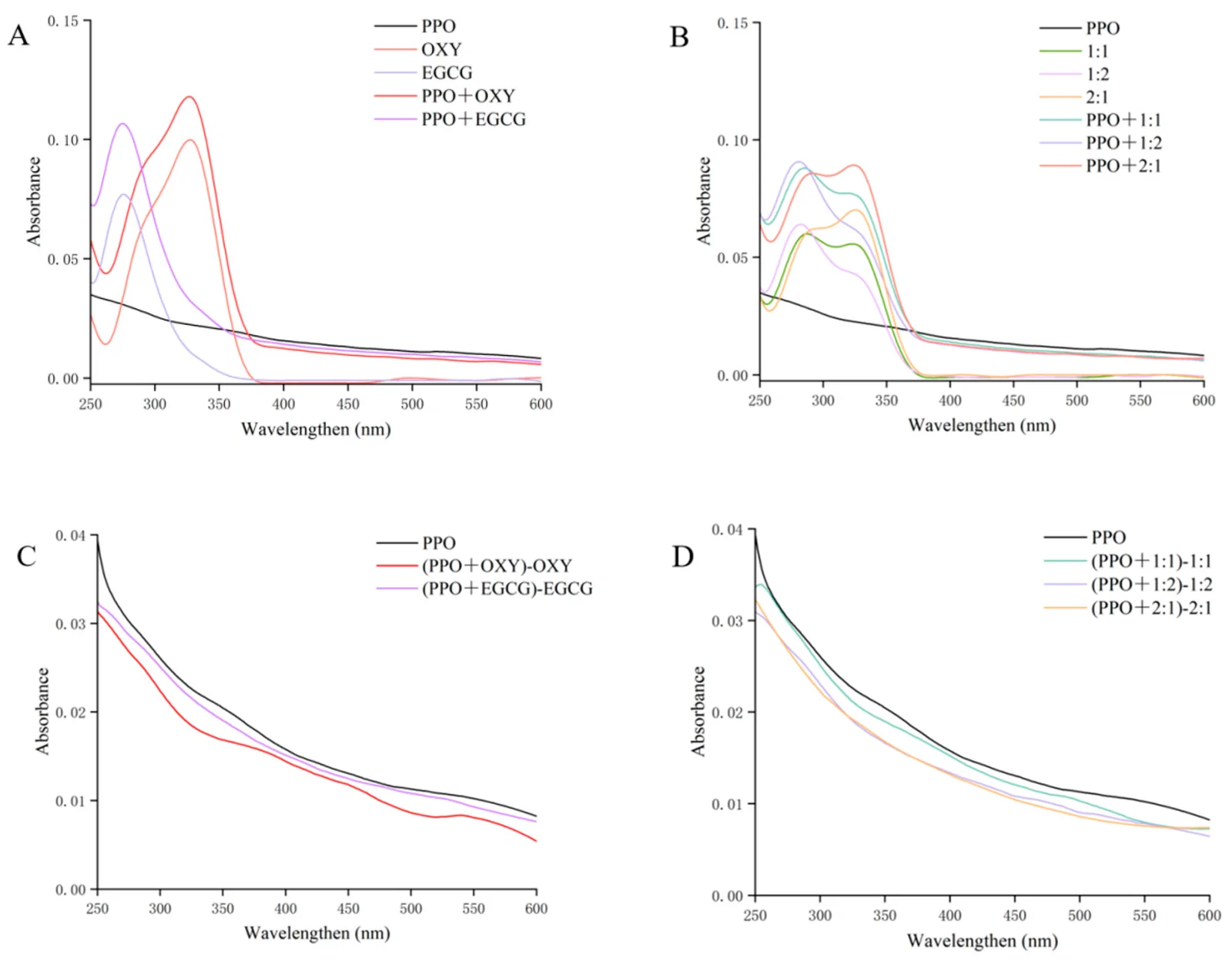
UV spectrum of OXY and EGCG combined treatment (**A**,**B**), and the spectrum of PPO after background subtraction of the inhibitor (**C**,**D**) (Among these, PPO, OXY, EGCG, 1:1, 1:2, 2:1 represent the UV spectra of untreated PPO solution and inhibitor-only solutions; PPO + OXY/EGCG/1:1/1:2/2:1 denotes the UV spectra of PPO solutions treated with corresponding inhibitors; (PPO + OXY/EGCG/1:1/1:2/2:1)-OXY/EGCG/1:1/1:2/2:1 represents the UV spectrum of PPO after removing the inhibitor’s own spectrum following treatment with the corresponding inhibitor).

UV-visible spectroscopy reflects the formation and conformational changes of protein-ligand complexes. Absorbance shifts at 250–300 nm correlate with alterations in aromatic residues such as tryptophan and tyrosine [20,35]. As shown in Figure 4A, OXY exhibited a characteristic peak at 325 nm, while EGCG showed a distinct peak at 270 nm. The addition of PPO induced overlapping effects at these wavelengths in both systems. When OXY and EGCG were co-administered at varying ratios (Figure 4B), both characteristic peaks were retained in the UV spectrum. Compared to other combined treatment groups, the 1:2 combined mixture exhibited the highest absorption peak at 250–300 nm, suggesting this treatment may expose more aromatic amino acids. Since aromatic rings could interact with the tyrosinase active site via π-π stacking, they interfere with enzyme–substrate binding, thereby inhibiting enzyme activity [20]. The EGCG content increased in the system, which caused strong molecular interactions, potentially exposing more chromophores and leading to an increase in absorbance [36].

Dynamic quenching occurs when quenchers collide with proteins, leaving UV spectra unchanged. In contrast, static quenching involves quenchers binding to proteins to form new complexes and alters UV spectra [37]. After subtracting the inhibitor background (Figure 4C,D), all treatments showed non-overlapping and significantly reduced curves compared to the native PPO. This suggested that the combined inhibitors formed stable ground-state complexes with PPO, which is consistent with a static quenching mechanism. The difference in absorbance between the PPO treated with a 1:2 and 2:1 ratio and the original enzyme was the greatest, indicating a greater impact of inhibitors on enzyme conformation. Ma et al. [38] found that UV spectra treated with different concentrations of EGCG exhibited varying degrees, which was similar with our findings. In addition, Wang et al. [37] and Zhang et al. [39] also found the conformation of tyrosinase was significantly changed by using pyrimidine-thiol and furanol derivatives.

### 3.5. Effects of OXY and EGCG on the Secondary and Tertiary Structures of PPO

**Figure 5:**
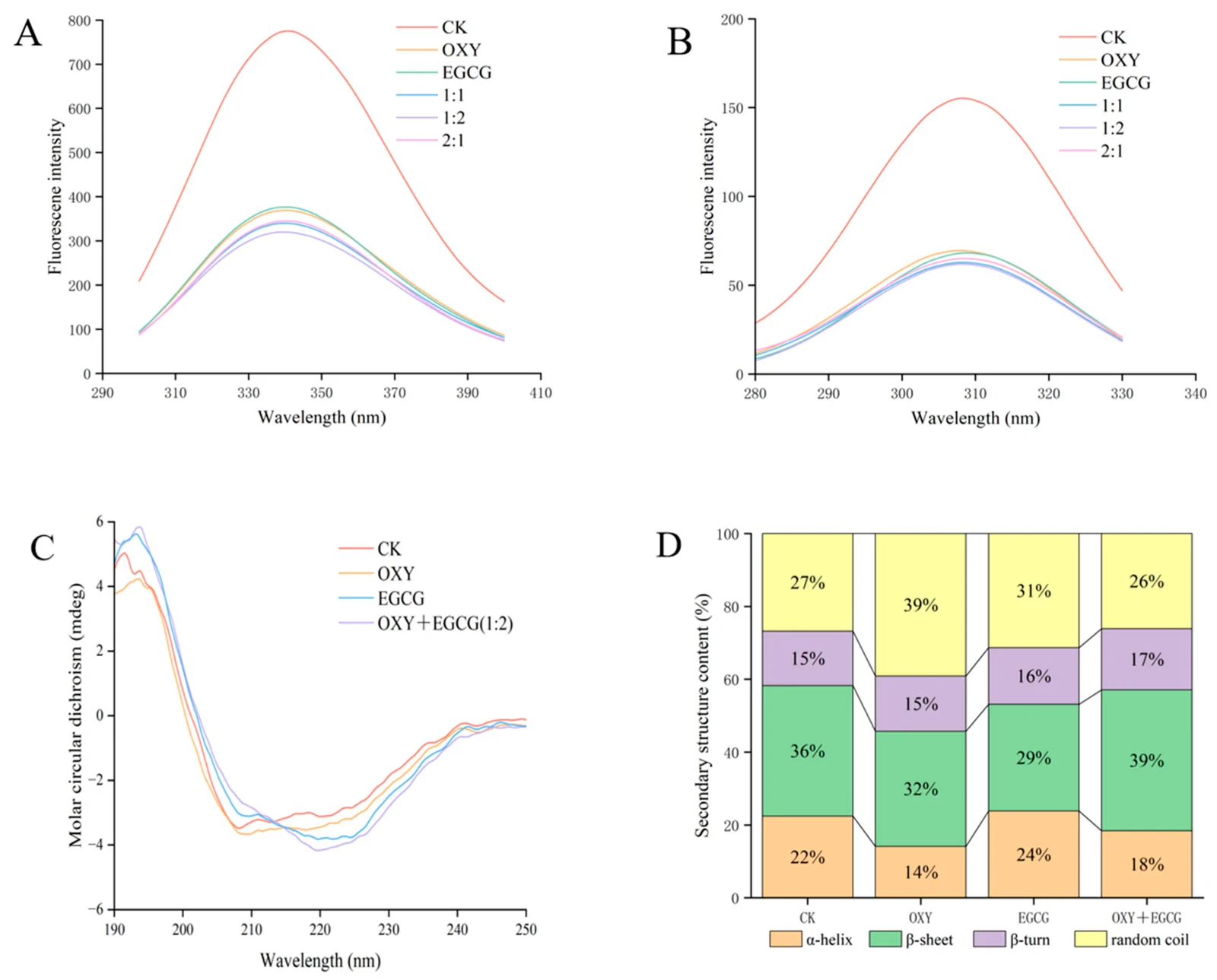
Effects of combined treatment with OXY and EGCG on the tertiary and secondary structures of PPO ((**A**,**B**) represent the fluorescence emission spectra and synchronous fluorescence spectra for OXY + EGCG combined treatment, respectively, Δλ = 15 nm; (**C**,**D**) represent the circular dichroism spectra and the specific proportions of secondary structures for OXY and EGCG, respectively, following combined treatment).

The intrinsic fluorescence of PPO originates from endogenous fluorophores such as tryptophan and tyrosine residues. Upon excitation at 280 nm, it emits characteristic fluorescence, and alterations in fluorescence intensity directly reflect the binding interaction with inhibitors and concomitant conformational changes [40,41]. As shown in Figure 5A, the maximum emission peak of native PPO’s intrinsic fluorescence occurred at 340 nm. The fluorescence intensity of PPO treated with OXY and EGCG (1:2 ratio) was significantly lower than that of the single-treatment groups, exhibiting an emission intensity of 42.00% relative to the native enzyme. These results indicated that the combined inhibitors bound to amino acid residues on the enzyme surface by abundant phenolic hydroxyl groups, thereby inducing PPO fluorescence quenching [42]. Furthermore, combined inhibitors may alter the local microenvironmental polarity of the enzyme, leading to decreased fluorescence intensity [43]. This quenching further implies that tryptophan residues might undergo denaturation post-treatment, exposing them to a more non-polar and hydrophobic environment [13]. In addition, the Δλ = 15 nm synchronous fluorescence spectrum reflects alterations in the microenvironment surrounding the tyrosine residue [44]. As shown in Figure 5B, the results also exhibited a trend similar to the emission fluorescence spectrum, which the 1:2 combined treatment group showed the lowest fluorescence intensity with only 39.81% of the native enzyme. These changes suggested that the tyrosine residues of PPO were exposed to a more hydrophobic environment [18]. Similar results were observed by Tian et al. [45] with a decrease in peak intensity of PPO when binding with EGCG. This could be attributed to altered polarity of the fluorophore after binding, effectively quenching fluorescence intensity. Furthermore, other studies also indicated that ergothioneine [44], apple pectin [46], and pyrimidine-2,4-dithiol [37] could disrupt enzyme tertiary structure via fluorescence quenching.

As shown in Figure 5C, the native enzyme exhibits two distinct characteristic peaks at 208 nm and 220 nm, corresponding to an α-helical conformation [13]. Regarding the specific proportions of secondary structures (Figure 5D), the native PPO exhibited α-helix, β-sheet, β-turn, and disordered coils accounting for 22.4%, 35.9%, 14.9%, and 26.8%, respectively. OXY treatment significantly reduced the ordered secondary structures of PPO, decreasing the α-helix content from 22.4% to 14.1% and the β-sheet content from 35.9% to 31.6%, while increasing the proportion of random coils to 39.1%. This marked increase in structural disorder indicates that OXY promotes conformational relaxation of PPO [34]. In contrast, EGCG induced a distinct structural response. The contents of α-helices and random coils increased to 23.8% and 31.3%, respectively, whereas β-sheet content decreased to 29.3%. These changes in secondary structures indicated varying degrees of PPO conformation alteration, consistent with the findings of Tian et al. [27] on PPO treated by EGCG inhibitor. Notably, under combined OXY + EGCG treatment, the most pronounced redistribution trend was toward β-type structures. The contents of β-sheets and β-turns rose to 38.7% and 16.8%, respectively, while α-helix content dropped to 18.4%, and random coils remained relatively low at 26.1%. These findings indicate that the combined treatment did not simply enhance the OXY-induced unfolding; rather, it promoted a unique β-enriched conformational rearrangement in PPO, which likely corresponds to the more pronounced perturbation of the enzyme’s structure observed in the CD spectra. It was speculated that the combined action of the two inhibitors altered the microenvironment near PPO, thereby influencing its secondary structure [47]. Furthermore, due to the differences in binding sites and modes among the inhibitors, the resulting conformational changes in PPO also varied [23].

### 3.6. Transmission Electron Microscopy (TEM) Observation

**Figure 6:**
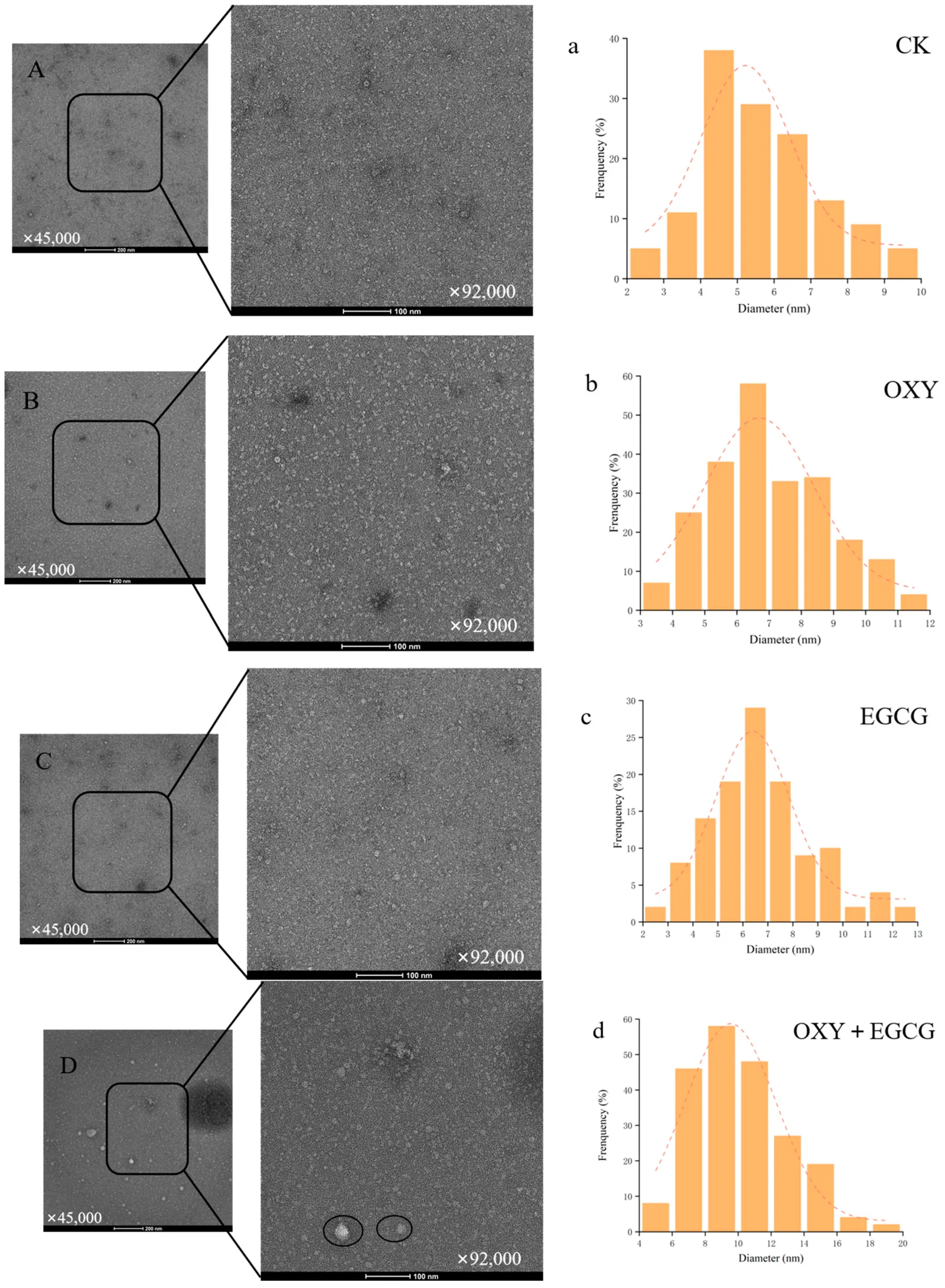
Transmission electron microscopy (TEM) images of PPO combined with OXY and EGCG treatments, and the statistical curves of particle size distribution fitted using the Gaussian equation ((**A**,**a**) phosphate buffer; (**B**,**b**) OXY; (**C**,**c**) EGCG; (**D**,**d**) OXY + EGCG (1:2)).

As shown in Figure 6A, the distribution of untreated PPO was relatively uniform, with particle diameters ranging from 2.20 to 9.54 nm and a peak size of 5.22 nm. However, after treatment with inhibitors, the PPO structure underwent significant changes: the boundaries of the PPO became blurred, and the distribution of PPO treated with OXY and EGCG was no longer uniform. Particle diameters increased due to enzyme aggregation, and the peaks of the particle size distributions rose to 6.69 nm and 6.39 nm, respectively (Figure 6B,C). When the two inhibitors were mixed in a 1:2 ratio (Figure 6D), the impact on the PPO structure became significantly greater. The PPO particle size increased significantly with a peak diameter of 9.53 nm accounting for approximately 23.30% of the total volume. With an average size of 10.11 nm, it was extremely significantly higher than the control and individual groups (*p* < 0.001), demonstrating a statistically supported combined effect of the mixed inhibitors on PPO aggregation. This finding is consistent with the results reported by Tian [13] and Du et al. [18], who treated PPO with EGCG and cold plasma, respectively, resulted in protein aggregation. Therefore, it was speculated that the combined treatment caused protein aggregation, thereby burying or occupying partial active sites of PPO and inhibiting its catalytic activity [45].

### 3.7. X-Ray Diffraction (XRD)

**Figure 7:**
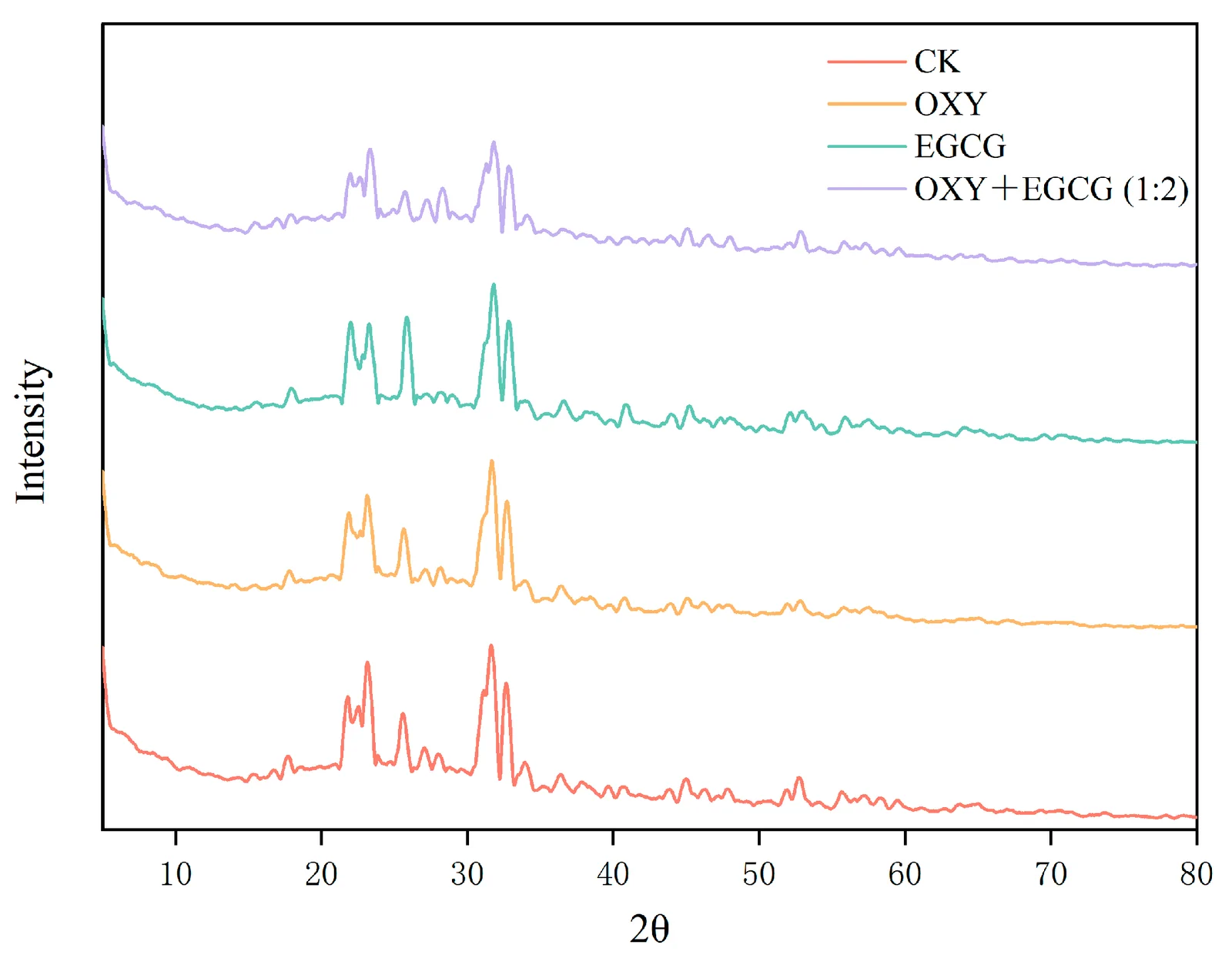
X-ray diffraction (XRD) images of PPO treated with different inhibitors OXY and EGCG.

In this study, the phosphate concentration, pH, lyophilization conditions, and testing parameters were strictly identical across all four groups, so the salt crystallization background should have remained approximately constant among groups. As shown in Figure 7, untreated PPO (CK) exhibited sharp diffraction peaks at 2θ = 23°, 25°, 31°, and 53°, which was generally consistent with the characteristic peak results reported by Jin et al. [19]. Compared to the native enzyme, the positions of the diffraction peaks did not show significant changes after combined treatment, but the peak intensities decreased to varying degrees. Among them, the combined treatment group exhibited the lowest peak intensity at the characteristic peaks, with the highest peak intensity (2θ = 31°) decreasing by 25.32%, 24.03%, and 19.58% compared to the native enzyme and the single-treatment groups with OXY and EGCG, respectively. This suggested that the combined treatment disrupted the PPO crystal structure, resulting in the lowest crystallinity [48]. The reduction in crystallinity suggested that the combined inhibitors successfully infiltrated the enzyme’s architecture, leading to structural disorder within the PPO [49]. Ahmed et al. [50] similarly observed a decrease in XRD peak intensity during their study of blanching yam polyphenol oxidase with high-humidity hot air treatment. Furthermore, the positions of the diffraction peaks are also related to changes in the crystal structure [51]. The addition of inhibitors caused the peak of maximum intensity to shift toward 2θ = 32°, with the combined treatment group exhibiting the greatest shift in crystal structure. Senthilkumar et al. [52] found that the XRD patterns of red kidney bean protein isolates exhibited peak intensity reduction and peak shifts following heat treatment, indicating that their native crystal structures and protein conformations were altered, which is consistent with our findings.

### 3.8. Application on Fresh-Cut Pear

**Figure 8:**
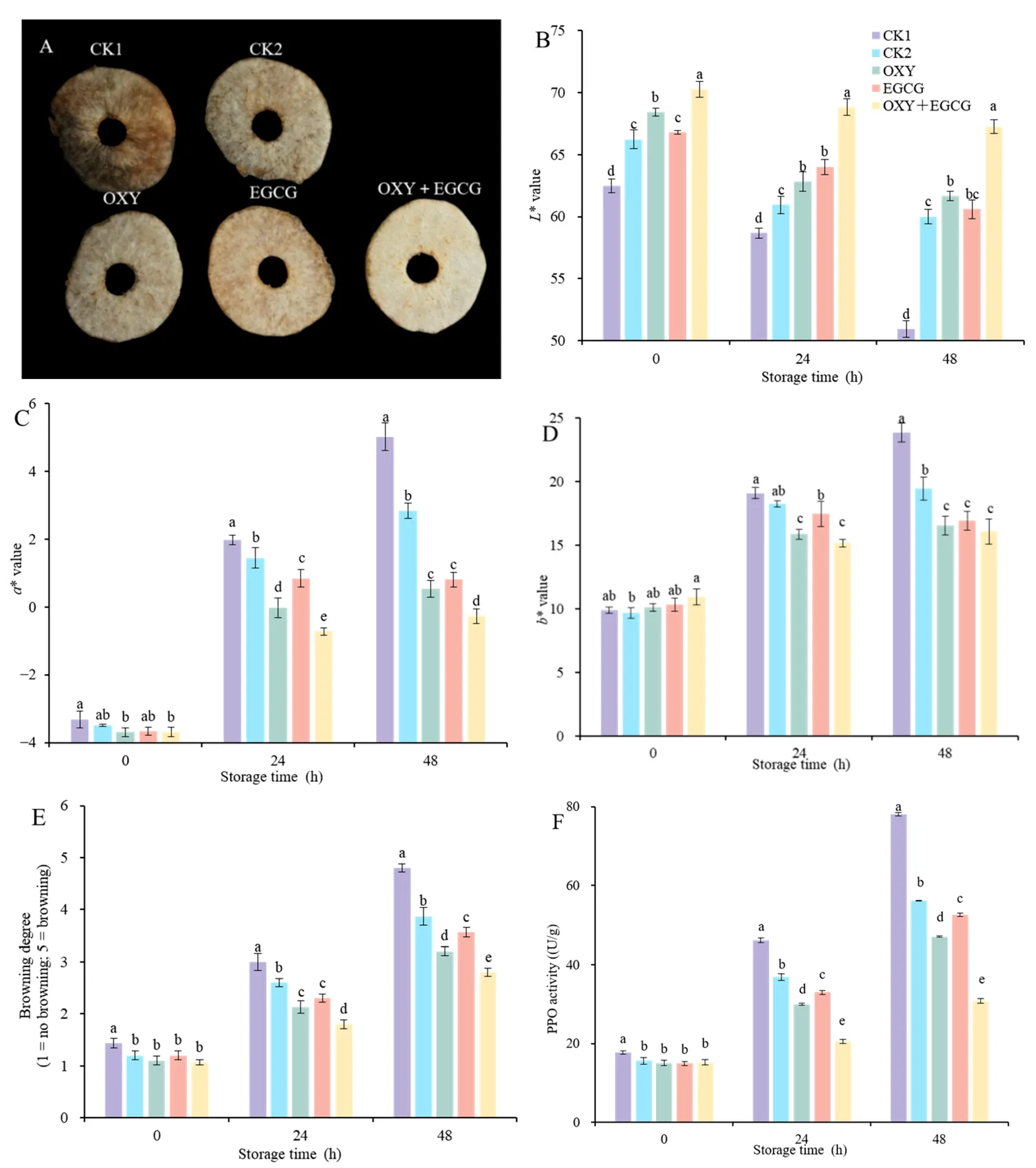
Effects of the combined treatment with OXY and EGCG on the appearance quality (**A**) of fresh-cut pear slices after 48 h of storage, as well as the surface color L* (**B**), a* (**C**), and b* (**D**) values, browning degree (**E**) and PPO activity (**F**) during storage. Different lowercase letters indicate significant differences among groups (*p* < 0.05).

To verify whether the findings could be applied to fresh-cut fruits and vegetables, fresh-cut pear slices were selected for effect verification. As shown in Figure 8A, after the 48 h of storage, all treatment groups exhibited better anti-browning effect on fresh-cut pear slices. Among them, the unsoaked control group (CK1) showed the most severe browning with uneven texture and localized dehydration, causing the pear slices to become soft and thin. The distilled water group (CK2) exhibited extensive browning on the surface and slight dehydration. In contrast, the EGCG-treated group exhibited a deep brown surface color distinct from the CK group, which presumably driven by EGCG oxidation. The OXY treatment showed better white than EGCG, but a small number of brown spots still appeared. The combined treatment group performed best and showed a uniform texture and high sensory quality without obvious browning and water loss. For L*, a*, and b* values (Figure 8B–D), with the increasing storage time, L* values showed a gradual downward trend, while a* and b* values showed a gradual upward trend. In contrast, the L* value of the combined treatment group decreased at the slowest rate. After 48 h of storage, the L* values of the combined treatment group were 32.06%, 12.14%, 9.10%, and 11.02% higher than those of the CK1, CK2, OXY, and EGCG single-treatment group, respectively. The a* and b* value of the combined treatment group increased at a slower rate. However, the effect of combined treatment on the b* value showed no significant difference compared to other single inhibitor group after 48 h of storage. The trends in color changes were similar to the results of He et al. [53] regarding the real-time prediction of freshness during the storage period of Korla pears. In addition, the browning degree in fresh-cut pear slices increased in all treatment groups during storage (Figure 8E), while the combined treatment group exhibited the lowest degree of browning and maintained good color and sensory quality. In addition, the PPO activity of fresh-cut pear slices showed an overall upward trend during the storage (Figure 8F). Compared with the control group, all treatment groups effectively inhibited the increase in PPO activity, with the OXY + EGCG group showing the best inhibitory effect. At the 48 h of storage, the OXY + EGCG group exhibited the lowest PPO activity, showing reductions of 60.52%, 45.12%, 34.46%, and 41.00% compared to the CK1, CK2, OXY, and EGCG groups, respectively. Men et al. [54] also found in their experiment using that α-lipoic acid effectively inhibited PPO activity of fresh-cut pears to suppress browning. In this study, the OXY and EGCG inhibited the increase in PPO activity, further corroborating the combined inhibitors could alter the conformation structure of PPO, thereby affecting its activity. In summary, the OXY + EGCG combined treatment was able to delay the decline in color and the browning degree in fresh-cut pear slices and inhibit the increase in PPO activity, demonstrating good potential for commercial preservation applications.

It should be noted that the storage assay was conducted under ambient conditions, and a 48 h storage period was selected to capture the early-stage browning dynamics. Additionally, a conventional inhibitor (e.g., ascorbic acid) was not included as a positive control in this study. Future work will extend storage durations under simulated commercial conditions and expand the model system to other browning-susceptible produce (e.g., button mushrooms, apples, potatoes, and lotus roots) to verify the broad-spectrum applicability of the combined effect.

## 4. Conclusions

This study demonstrated that the combination of oxyresveratrol (OXY) and epigallocatechin gallate (EGCG) significantly inhibited polyphenol oxidase (PPO) activity. The combined effect was optimal at a 1:2 ratio, with a combined coefficient of 1.27. Kinetic analysis indicated that this combined inhibition represented a mixed-type inhibition, which acted simultaneously on free PPO and enzyme–substrate complexes to block enzymatic reactions. Mechanistically, the combined treatment formed a more stable OXY-EGCG-PPO ternary complex, achieving efficient inhibition by altering PPO’s secondary and tertiary structures and disrupting its active site conformation. Additionally, the combined treatment induced apparent protein aggregation and weakened the intensity of crystal diffraction peaks. When applied to fresh-cut pear slices, the combined system effectively delayed enzymatic browning and maintained desirable sensory quality. These findings obtained from fresh-cut pear flesh provide a theoretical basis and practical potential for the application of OXY and EGCG as a natural combined anti-browning technology in the fresh-cut produce industries.

## Figures and Tables

**Table 1:** The combined coefficients of catechol and levodopa as substrate.

	Substrate	Catechol	Levodopa
Combined Ratio ^1^			
1:1		1.26	1.55
1:2		1.27	1.68
1:3		1.24	1.64
1:4		1.21	1.46
2:1		1.10	1.12
3:1		0.91	1.09
4:1		0.84	1.00

^1^ This combined ratio refers to the ratio of oxyresveratrol to epigallocatechin gallate (OXY: EGCG).

**Table 2:** Summary table of molecular docking binding energies and hydrogen-bonding residues.

Ligand		Amino Acid Residue	Bonding Length (Å)	Binding Affinity (Kcal/mol)
OXY		Gln-41	3.0	−8.09
		Glu-173	2.6	
		Lys-180	3.1	
			2.3	
EGCG		Glu-67	2.6	−10.41
		Lys-70	2.8	
		Tyr-62	2.1	
OXY + EGCG	OXY	Glu-340	2.7	−16.63
		Tyr-343	2.2	
		Gln-74	2.3	
		Ile-96	2.7	
			3.1	
	EGCG	Glu-67	2.3	
		Lys-70	2.6	
		Tyr-62	2.5	
		Gln-74	3.4	
			3.5	

## Data Availability

The original contributions presented in the study are included in the article, and further inquiries can be directed to the corresponding author.

## References

[1] Shi J., Xie W., Zhang K., Tian X., Zhao Z., Liu L., Li S., Li Q. (2026). Curcumin inhibits enzymatic browning in fresh-cut potatoes through regulating membrane stability and SA signaling. Innov. Food Sci. Emerg. Technol..

[2] Latifi-Amoghin M., Abbaspour-Gilandeh Y., De La Cruz-Gámez E., Hernández-Hernández M., Hernández-Hernández J.L. (2025). Nondestructive Detection of Polyphenol Oxidase Activity in Various Plum Cultivars Using Machine Learning and Vis/NIR Spectroscopy. Foods.

[3] Zhang S., Lin Y., Zeng X., Liu G., Ye G., Du C., Li Y., Lin W., Wei K., Li Z. (2025). Genome-wide characterization of polyphenol oxidase (PPO) gene family in tobacco and the molecular improvement of browning reaction for K326 tobacco leaves. Ind. Crops Prod..

[4] Zhou X., Xiao Y., Meng X., Liu B. (2018). Full inhibition of Whangkeumbae pear polyphenol oxidase enzymatic browning reaction by L-cysteine. Food Chem..

[5] Zheng J., Zhang M., Zhao Z., Li M., Kang J., Lu L., Qiao L., Liu X. (2026). Niacin Alleviates Browning in Fresh-Cut Potatoes: Regulation of NADPH/NADH Levels Mediates ROS-Redox Homeostasis and the Ascorbate–Glutathione Cycle. Foods.

[6] Ebrahimi P., Lante A., Grossmann L. (2025). Protein-polyphenol complexation vs. conjugation: A review on mechanisms, functional differences, and antioxidant-emulsifier roles. Food Hydrocoll..

[7] Niu B., Fei Y., Liu R., Chen H., Fang X., Wu W., Mu H., Gao H. (2023). Effect of oxyresveratrol on the quality and membrane lipid metabolism of shiitake mushroom (*Lentinus edodes*) during storage. Food Chem..

[8] Zhang B., Fu J., Wang C., Yin M., Liu P., Zhang S., Zhang X., Peng Y. (2025). Inhibitory effect of oxyresveratrol on the browning and PPO of fresh-cut potato. Postharvest Biol. Technol..

[9] Zeng H., Li Q., Ma J., Yang R., Qu L. (2021). A comparative study on the effects of resveratrol and oxyresveratrol against tyrosinase activity and their inhibitory mechanism. Spectrochim. Acta A.

[10] Yin Z., Li Y., Gan H., Feng N., Han Y., Li L. (2022). Synergistic effects and antityrosinase mechanism of four plant polyphenols from Morus and Hulless Barley. Food Chem..

[11] Shi J., Gong Z., Cao Z., Hou F., Cui W., Jia F., Jiao J., Zhou Q., Wang W., Wang Y. (2025). Mechanism of oxalic acid delaying browning of fresh-cut apples mediated by synergistic regulation of phenol metabolism and oxidative stress. Postharvest Biol. Technol..

[12] Nirmal N.P., Benjakul S. (2009). Melanosis and quality changes of pacific white shrimp (*Litopenaeus vannamei*) treated with catechin during iced storage. J. Agric. Food Chem..

[13] Song X., Ni M., Zhang Y., Zhang G., Pan J., Gong D. (2021). Comparing the inhibitory abilities of epigallocatechin-3-gallate and gallocatechin gallate against tyrosinase and their combined effects with kojic acid. Food Chem..

[14] Tian X., Li Y., Ma T., Lv Y., Wang Z., Wang Y., Liao X. (2025). A strategy to significantly improve the efficacy of high hydrostatic pressure in inactivating polyphenol oxidase using epigallocatechin gallate and ferulic acid for the inhibition of enzymatic browning: From a model system to cloudy apple juice. Food Chem..

[15] Tallarida R.J. (2011). Quantitative Methods for Assessing Drug Synergism. Genes Cancer.

[16] Xu Y., Xiao S., Liu Y., Wang Y., Hou S., Teng J. (2025). The stability of polyphenol oxidase in deep eutectic solvents: Insights from spectroscopy and molecular docking. Int. J. Biol. Macromol..

[17] Cheng D., Wang G., Tang J., Yao C., Li P., Song Q., Wang C. (2020). Inhibitory effect of chlorogenic acid on polyphenol oxidase and browning of fresh-cut potatoes. Postharvest Biol. Technol..

[18] Du Y., Huang X., Yuan S., Yu H., Guo Y., Yuliang C., Yao W. (2025). Cold plasma and honey synergistically inhibit polyphenol oxidase to enhance fresh-cut apple preservation. Food Chem..

[19] Jin J., Deng X., Zhou J., Deng Y., Pan N., Luo Y., Awwal M. (2025). The mechanisms of inactivation of polyphenol oxidase in fresh-cut *Agaricus bisporus* by dual-frequency ultrasound combined with electrolytic water. Ultrason. Sonochem..

[20] Guo C., Yang B., Wang J., Wen T., Jin X., Li M., Yu L., Zhang T. (2025). Identification of millet bran protein-derived peptides as natural tyrosinase inhibitors: Preparation, mechanism of action and anti-browning application. Int. J. Biol. Macromol..

[21] Song Z., Qiao J., Tian D., Dai M., Guan Q., He Y., Liu P., Shi J. (2023). Glutamic acid can prevent the browning of fresh-cut potatoes by inhibiting PPO activity and regulating amino acid metabolism. LWT.

[22] Zhang S., Wang S., Li Y., Wang J., Shi J., Peng Y., Liu P. (2025). Ursolic acid, a natural endogenous compound, inhibits browning in fresh-cut apples. Postharvest Biol. Technol..

[23] Chang X., Wang Q., Dong T., Feng Y. (2025). The role of chicory furaneol in reducing potato browning: Inhibiting polyphenol oxidase activity and enhancing antioxidant capacity. Postharvest Biol. Technol..

[24] Zhao J., Yao G., Chen J., Zhu L. (2025). Characterisation of pullulan polysaccharide membranes cross-linked with epigallocatechin gallate and Fe^3+^ for passion fruit preservation. Food Chem. X.

[25] Nam S.H., Bharti D., Seong H.J., Kim Y.M., Jeong C., Yang K.Y. (2024). Nanoemulsification of epigallocatechin gallate for debitterization, antibrowning, and inhibition of intracellular lipid accumulation. Food Biosci..

[26] Li G., Wang X., Zhu H., Li G., Du J., Song X., Erihemu (2023). Use of different food additives to control browning in fresh-cut potatoes. Food Sci. Nutr..

[27] Tian X., Rao L., Zhao L., Wang Y., Liao X. (2022). Multispectroscopic and computational simulation insights into the inhibition mechanism of epigallocatechin-3-gallate on polyphenol oxidase. Food Chem..

[28] Sae-Leaw T., Benjakul S., Simpson B.K. (2017). Effect of catechin and its derivatives on inhibition of polyphenoloxidase and melanosis of Pacific white shrimp. J. Food Sci. Technol..

[29] Zhang S., Liu H., Liu J., Qin L., Li X., Wei X., Chen X., Wang L. (2026). Polyphenol oxidase-mediated browning during storage of *Pinus elliottii* oleoresin: Characterization of pine-needle PPO and antibrowning action of L-cysteine. Food Biosci..

[30] Yu Q., Fan L., Duan Z. (2019). Five individual polyphenols as tyrosinase inhibitors: Inhibitory activity, synergistic effect, action mechanism, and molecular docking. Food Chem..

[31] Assaggaf H., El Hachlafi N., Elbouzidi A., Taibi M., Benkhaira N., El Kamari F., Alnasseri S.M., Laaboudi W., Bouyahya A., Ardianto C. (2024). Unlocking the combined action of *Mentha pulegium* L. essential oil and Thym honey: In vitro pharmacological activities, molecular docking, and in vivo anti-inflammatory effect. Heliyon.

[32] Chen S., Zhou Y., Sun P., Sun S., Shao P. (2026). Unveiling the anti-melanogenic effect of the “Nine Steaming Nine Sun-Drying” processed *Polygonatum sibiricum* saponins: A synergy of network pharmacology and molecular docking. Food Biosci..

[33] Du Y., Zhao L., Yuan Z., Zhang Y., Chen Y., Ding Y., Jiao X., Zhao C., Shimizu K., Yang B. (2026). Molecular mechanism of synergistic tyrosinase inhibition by blueberry and black chokeberry anthocyanidins: Optimal synergistic formulation for multi-berry beverage development. Food Chem. X.

[34] Liu L., Li J., Zhang L., Wei S., Qin Z., Liang D., Ding B., Chen H., Song W. (2022). Conformational changes of tyrosinase caused by pentagalloylglucose binding: Implications for inhibitory effect and underlying mechanism. Food Res. Int..

[35] Li J., Ni Y., Li J., Fan L. (2024). Unveiling the synergistic inhibition mechanism of polyphenols in Flos Sophorae Immaturus tea on xanthine oxidase by multi-spectroscopy, molecular docking and dynamic simulation methods. J. Mol. Liq..

[36] Xu G., Zhao J., Shi K., Pan S. (2025). Aging alleviates the negative impact of citrus peel polysaccharides on the inhibitory effect of EGCG against α-Amylase. Food Hydrocoll..

[37] Wang G., Zhang J., Peng Z. (2025). Tyrosinase inhibitory mechanism of pyrimidine-thiols and their potential application in the anti-browning of fresh-cut apples. Food Chem..

[38] Ma S., Liu X., Wang F., Chen L., Yuan M., Jiang Y., Zhao L., Bai C. (2026). Synergistic antioxidant action and enhanced digestive utilization of *Corbicula fluminea* protein enabled by binding to epigallocatechin gallate. LWT.

[39] Zhang J., Peng Z., Wang G. (2026). Design and synthesis of novel furaneol derivatives with fragrance as tyrosinase inhibitors for inhibiting the browning of fresh-cut banana. Food Chem..

[40] Pang H., Jia Y., Zhang Z., Xie Y., Song M., Cui B., Ye P., Chen X., Fu H., Wang Y. (2024). Mushroom polyphenol oxidase inactivation kinetics and structural changes during radiofrequency heating. Food Biosci..

[41] Fan W., He S., Ren H., Pan L., Liu L., Tian Y. (2026). Preparation and application of ergothioneine liposomes and their inhibitory effect on polyphenol oxidase from Lanzhou lily. Food Biosci..

[42] Hong X., Luo X., Wang L., Gong D., Zhang G. (2023). New Insights into the Inhibition of Hesperetin on Polyphenol Oxidase: Inhibitory Kinetics, Binding Characteristics, Conformational Change and Computational Simulation. Foods.

[43] Chai Z., Yang B., Cheng J., Xi P., He N., Kang K. (2026). Synergistic inactivation of lycium polyphenol oxidase by acidic electrolyzed water combined with vacuum pulsed drying: Mechanism and color retention study. Food Biosci..

[44] Ma Y., Ma Y., Wang S., Zhai Y., Xiang Q., Li X. (2025). Elucidating the inhibitory activity and mechanism of ergothioneine on polyphenol oxidase from mushroom. Int. J. Biol. Macromol..

[45] Tian Y., Lv Y., Zhao L., Wang Y., Liao X. (2024). Insight into the mechanism of high hydrostatic pressure effect on inhibitory efficiency of three natural inhibitors on polyphenol oxidase. Food Chem..

[46] Zhang S., Jiao W., Ni C., Hao G., Huang M., Bi X. (2025). Effect of pectin on the inactivation of mango polyphenol oxidase treated by high-intensity ultrasound. LWT.

[47] Shen H., Zhao M., Sun W. (2019). Effect of pH on the interaction of porcine myofibrillar proteins with pyrazine compounds. Food Chem..

[48] Gholami A.A., Taghizadeh M.S., Moghadam A., Afsharifar A., Niazi A. (2026). Unraveling structure–function relationships and antioxidant stability of *Ferula assafoetida* seed protein hydrolysates with physicochemical and sensory characterization. J. Agric. Food Res..

[49] Ali K., Liu C., Niaz N., Haq F.U.I., Xu M., Sun R., Zhu T., Yin P., Wu M. (2026). Pea protein isolate-sodium alginate-based plant-based meat analogue: Dual crosslinking and mo insight mechanisms. Food Res. Int..

[50] Ahmed J.M.A., Mowafy S., Guo J., Okaiyeto S.A., Yu S., Liu Y. (2026). High humidity hot air impingement blanching (HHAIB) for controlled polyphenol oxidase inactivation, antinutrient reduction, and structural quality preservation in yam. Food Control.

[51] Yang J., Wu L., Chen X., Zhang J., Lian Y., Ji P., Chen C., Dou S. (2026). Correlation analysis between diffraction peak shift and broadening based on SWXRD, and the microstructure and multi-scale stress of 7A52 aluminum alloy. Mater. Charact..

[52] Senthilkumar N.V.K., Govindarajan N.N., Pandiselvam R., Kambhampati V., Sivaraj D. (2026). Comparative characterization of protein isolates from treated and un-treated Red kidney bean (*Phaseolus vulgaris*) and horsegram (*Macrotyloma uniflorum*) with commercial pea protein (*Pisum sativum*): Nutritional, functional, in vitro digestibility and structural perspectives. Food Chem..

[53] He Z., Yu J., Zhou X., Tang T., Wang H., Gong J., Shi J., Zhang X., Zhao Y. (2025). Multi-sensor fusion combined with machine learning enables real-time freshness prediction of ‘Korla’ pear during storage. Postharvest Biol. Technol..

[54] Men C., Wu C., Zhang Y., Wang M., Chen C., Liu L., Zheng L. (2024). α-Lipoic acid treatment regulates enzymatic browning and nutritional quality of fresh-cut pear fruit by affecting phenolic and carbohydrate metabolism. Food Chem..

